# Fusion Transcripts of Adjacent Genes: New Insights into the World of Human Complex Transcripts in Cancer

**DOI:** 10.3390/ijms20215252

**Published:** 2019-10-23

**Authors:** Vincenza Barresi, Ilaria Cosentini, Chiara Scuderi, Salvatore Napoli, Virginia Di Bella, Giorgia Spampinato, Daniele Filippo Condorelli

**Affiliations:** Department of Biomedical and Biotechnological Sciences, Section of Medical Biochemistry, University of Catania, 95123 Catania, Italy; ilaria.cosentini.93@gmail.com (I.C.); scuderi331@gmail.com (C.S.); napo.salvo@gmail.com (S.N.); virgi.db95@hotmail.it (V.D.B.); giorgiaspampinato@unict.it (G.S.)

**Keywords:** FuTAG, Cis-SAGe, readthrough, fusion transcript, alternative transcription, cancer

## Abstract

The awareness of genome complexity brought a radical approach to the study of transcriptome, opening eyes to single RNAs generated from two or more adjacent genes according to the present consensus. This kind of transcript was thought to originate only from chromosomal rearrangements, but the discovery of readthrough transcription opens the doors to a new world of fusion RNAs. In the last years many possible intergenic cis-splicing mechanisms have been proposed, unveiling the origins of transcripts that contain some exons of both the upstream and downstream genes. In some cases, alternative mechanisms, such as trans-splicing and transcriptional slippage, have been proposed. Five databases, containing validated and predicted Fusion Transcripts of Adjacent Genes (FuTAGs), are available for the scientific community. A comparative analysis revealed that two of them contain the majority of the results. A complete analysis of the more widely characterized FuTAGs is provided in this review, including their expression pattern in normal tissues and in cancer. Gene structure, intergenic splicing patterns and exon junction sequences have been determined and here reported for well-characterized FuTAGs. The available functional data and the possible roles in cancer progression are discussed.

## 1. Introduction

It is known that in human genomes the number of genes is significantly lower than the number of transcripts, due to a set of mechanisms such as alternative splicing, alternative promoter usage, alternative transcription termination sites, RNA editing, post-transcriptional alterations and so on. These mechanisms converge in the so-called genome complexity [1].

Sometimes these phenomena can lead to the production of transcript fusions, derived by transcription of two or more genes in a single RNA strand, with the possible formation of a fusion protein. Several known fusion transcripts are the results of chromosomal rearrangements and we can distinguish these transcripts as due to an intra- or an inter-chromosomal rearrangement. However, other fusion transcripts, not generated by genomic DNA alterations, have been described. They are formed at the RNA level and two main mechanisms have been suggested: Cis-splicing and trans-splicing [2,3].

Cis-fusion transcripts, such as transcription-induced chimeras (TIC) [4], tandem RNA chimeras [5], transcription-induced gene fusions (TIGF) [6] and readthrough transcripts and cis-splicing between adjacent genes (cis-SAGes) [7] are obtained from sequential transcription of adjacent genes, which lie on the same chromosome, the same DNA strand and with the same orientation. A single primary transcript (pre-RNA) is formed by the two (or more) adjacent genes that undergo cis-splicing in order to obtain the mature transcript. Trans-fusion transcripts occur when two separate pre-RNA transcripts are spliced together by trans-splicing [3].

In other words, cis-fusion transcripts (also called cis-SAGes) are characterized by the intergenic splicing of the fusion pre-RNA transcribed from adjacent genes. Transcription is the first step in gene expression, in which a DNA segment is copied into mRNA through the RNA-polymerase, an enzyme able to bind to a specific DNA sequence, called promoter. The promoter guides the RNA-polymerase to identify the transcription start site and to initiate the RNA synthesis. In the majority of cases, transcription ends at a regulated termination point to avoid RNA-polymerase from transcribing through the next gene. The space between adjacent genes, called the intergenic region, generally is not transcribed into pre-mRNA. During cis-SAGe formation, the termination signal is ignored and the intergenic region is transcribed in the pre-mRNA and then spliced out as an intron with an intergenic splicing [8]. The conditions necessary for cis-SAGe formation are: The active transcription of the upstream gene, the transcriptional detour of gene transcriptional boundaries, the formation of a single pre-RNA containing sequences from both genes and the intergenic region and the production of a spliced mRNA containing exons from both genes. Dysregulation of the molecular machinery involved in the aforementioned steps influences the synthesis of the cis-SAGes [3].

However, the evidence for a cis-splicing mechanism in the formation of fusion transcripts of adjacent genes is not always compelling and in many cases the mechanism is only assumed [9]. Moreover, an unambiguous proof is technically demanding, considering that transcription and splicing often occur simultaneously or in a short interval. Different points of view on the nomenclature for fusion transcripts have been reported in the literature [3,9]. It has been suggested to reserve the term "fusion RNA" only for transcripts transcribed from fusion genes at the DNA level and to use the term "chimeric RNA" for transcripts derived by trans-splicing of two different pre-RNAs [9]. On the other hand, the terms "cis-Splicing of Adjacent Genes (cis-SAGe)" [3,10] or "gene readthrough" have been suggested for fusion transcripts deriving from adjacent genes through the readthrough and cis-splicing mechanisms. In this review we will focus only on fusion transcripts not associated to DNA structural abnormalities and involving adjacent genes. Since in the several cases the exact mechanism of generation of those transcripts is not known, we prefer to use the generic term fusion transcripts of adjacent genes (FuTAGs), without taking into account the mechanism of formation. These fusion transcripts are also categorized as intrachromosomal-single strand-0 gap [11]. 

## 2. The History of FuTAGs

In the last decade, several groups attempted to estimate the number of FuTAGs in the human genome. Akiva et al. [8] were among the first authors to investigate the human FuTAGs and to describe their structures and synthesis mechanisms. They have identified 212 cases of FuTAGs aligning to the entire genome, ESTs and cDNA sequences available in GenBank. 412 single genes are involved in the formation of FuTAGs and four of these contributed to multiple FuTAGs. Twenty of these were tested by using RT-PCR on different tissues and cell lines. Independently, Parra et al. [4] identified 127 FuTAGs by comparing ESTs and human genes sequences available on public databases and tested in RT-PCR [4]. Only thirteen of 127 cases are coincident with those reported by Akiva et al. [8].

**Figure 1:**
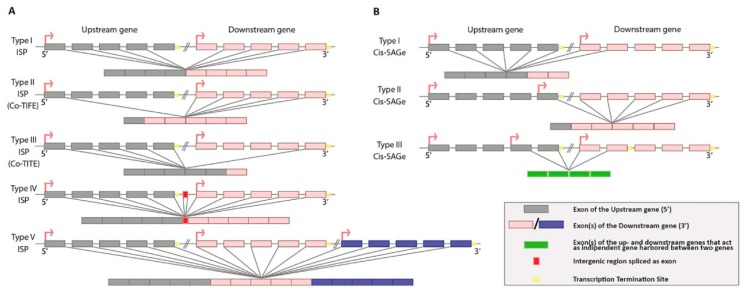
Schematic representation of different fusion transcripts of adjacent genes (FuTAGs) structures according to Lu et al. [12] and Yuan et al. [9] shown, respectively, in (**A**) and (**B**).

Generally, the resulting fusion transcript can hold some or all exons of two adjacent genes, in which the start site belongs to the upstream gene, while the termination site belongs to the downstream gene (Figure 1). In this condition, the intergenic region is removed, but new exon/s could be added due to the presence of splicing sites. The first hypothesis on these splicing mechanisms, called intergenic splicing patterns (ISPs), was postulated by Akiva et al. on the basis of their findings [8]. However, a recent classification of ISPs was formulated by Lu et al. [12], suggesting five possible ISPs involved in the formation of fusion transcripts (Figure 1A). Type I ISP is the most common one and consists in the detour of the termination site at the 3’-UTR of the upstream gene, so that transcription proceeds along the downstream gene. Grosso et al. [13], on the basis of RNAseq data, noticed that a transcription termination defect causes the bypass of the terminal 3’ splicing site (ss), thus the terminal 5’ ss of the upstream gene splice out with the 3’ ss of the downstream gene, thus excluding the last exon of the upstream gene and the first exon of the downstream gene. Type II ISP occurs between the first exon of upstream gene and any exon of the downstream gene and it is also known as co-transcription-induced first exon (Co-TIFE): The first exon usually contains regulatory motifs; thus, the upstream gene is capable of regulating the expression of the downstream gene transcriptionally (by promoters) and translationally (by 5’-UTR). Type III is like the Type II ISP, but in reverse: It contains the last exon of the downstream gene, it is also known as co-transcription-induced terminal exon (Co-TITE) and plays an important role in the efficacy of transcription termination and stability of the mRNA. Type IV ISP transcript contains novel exons obtained by the integration of the intergenic region. Finally, Type V ISP is generated by more than two parental genes [12].

In addition, Wen et al. [14] revealed that some FuTAGs could not originate only from adjacent genes in the same orientation, as described by Akiva et al. and Prakash et al., but also from parental genes with different orientation patterns. They identified FuTAGs showing a peculiar 3’-3’ orientation (tail to tail).

Yuan et al. [9] have categorized FuTAGs in three types (Figure 1B), considering the FuTAG: 1) As a splicing variant of the upstream gene; 2) as a splicing variant of the downstream gene, starting the transcription from an alternative start codon; and 3) as a canonical mRNA produced by a third gene harbored between the two genes, sharing exons with both and deemed as readthrough.

However, Yuan et al. [9] argue that a transcript generated by the cis-splicing of a novel pre-RNA should not be defined as a chimeric transcript but as the product of a novel gene overlapped to the previously known adjacent genes. Moreover, in a large number of fusion transcripts derived by adjacent genes a short homologous sequence (SHS) has been observed [9,15]. The presence of such SHS has also suggested another mechanism, called transcriptional slippage, that does not require the transcription of the intergenic region in the pre-mRNA [15]. Moreover, the possibility of RT-PCR artifacts in the generation of such transcripts has been discussed by Yuan et al. [9].

## 3. Functions of FuTAGs

The functional role of FuTAGs is unclear. There are only few examples of FuTAGs whose function is known. cis-SAGe can encode a protein containing coding sequences of both genes and might create a bifunctional protein with features from the two original proteins. The TWE-PRIL FuTAG (chromosome 17p13.1) is produced by the *TWEAK* gene (type-II transmembrane protein) and *APRIL* gene (secreted protein); both members, belonging to the TNF (Tumor Necrosis Factor) ligand family, are involved in angiogenesis signaling pathway and immune regulation. The TWE-PRIL transcript, revealed in human monocytes, primary T cells and in colorectal cell lines, is translated into a fused protein which comprises the TWEAK cytoplasmic and transmembrane domains combined with the APRIL C-terminal domain, which acts as a receptor binding domain [16]. Thus, TWE-PRIL and APRIL can recognize the same receptor allowing TWE-PRIL to be involved in cell–cell contact [16]. To date, this FuTAG has been renamed TNFSF12-TNFSF13. Details are reported in Table 1.

FuTAGs can change the features of the fused protein in relation to the parental genes. An example of this condition is Kua-UEV1 (also known as TMEM189-UBE2V1) that encodes a two-domain protein containing the Kua domain at the amino terminal and the UEV1 domain at carboxy terminal. The two parental genes, *Kua* and *UEV1*, located on chromosome 20q13.2, create a readthrough transcript comprising the first five exons of *Kua*, connected to the three exons of *UEV1*, removing exon 6 and 1 of *Kua* and *UEV1*, respectively (Type I ISP, Table 1). The UEV1 is a nuclear protein involved in the modulation of c-FOS activity, playing a crucial role in abnormal growth in human colon cancer cells; surprisingly the two-domain protein Kua-UEV is located in the cytoplasm as the wild type Kua protein. A consequence of the chimeric protein in the extranuclear compartment is its ability to polyubiquitinate specific proteins, or misfolded endoplasmic reticulum-associated proteins in the cytosol substrates [17,18]. Details are reported in Table 1.

Moreover, it has been observed that the production of FuTAG could be a possible mechanism that induces the upregulation of the downstream gene, like the anti-apoptotic oncogene *BCL2*, upregulated when the upstream gene *KDSR* undergoes readthrough transcription [13]. 

When the fusion phenomenon produces a reading frame-shift and the formation of a premature stop codon, the expression of the upstream gene can be suppressed by nonsense mediated decay (NMD); indeed, if the stop codon lies more than 50 nucleotides upstream of the final intron position, mRNA is recognized as nonsense and is degraded [19].

Some research groups have wondered what is the role of FuTAGs in pathology, noticing the increase of readthrough transcription in stressful conditions, such as heat shock, osmotic stress [20], oxidative stress and infection [21]. It has been hypothesized that there is a correlation between FuTAGs formation and cell aging, but the lack of a strong statistical significance dismissed this hypothesis [22].

## 4. Databases for Fusion Transcripts

In the last years the availability of advanced tools, such as microarray and NGS (Next Generation Sequencing) technologies, has improved the detection of FuTAGs; Kumar et al. reported a list of computational tools used to detect FuTAGs, such as EricScript and SOAPfuse [23]. These are computational frameworks, consisting in algorithms for the discovery of gene fusions in paired end RNAseq data. 

**Figure 2:**
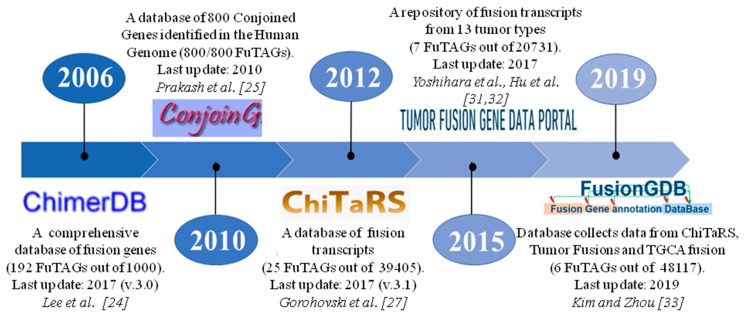
The timeline of five public databases collecting FuTAGs reports the year of publication, last update and number of FuTAGs compared to total records.

Nowadays there are five databases containing repositories of known cis- and trans- fusion transcripts (Figure 2). ChimerDB, built in 2006, was one of the first knowledge bases for fusion transcripts. Currently, it is at its third version composed by three modules: ChimerKB, ChimerPub and ChimerSeq. ChimerKB is a curated database containing more than 1000 fusion genes, of which 192 are FuTAGs; ChimerPub is a repository of fusion genes obtained by text mining of PubMed abstracts; finally, ChimerSeq archived more than 40,000 candidates obtained from deep-sequencing data from TCGA, without distinguishing cis/trans or intra/inter chromosomal rearrangements [24].

The first comprehensive database on FuTAGs was built by Prakash et al. [25] and called ConjoinG. The database collects information about FuTAGs, allowing visualization of mRNAs and ESTs, referring to adjacent genes in their genomic context. The FuTAGs listed in the ConjoinG database are the result of the alignments of mRNA and EST sequences of known genes to the entire human genome using the algorithm Conjoin, capable of recognizing FuTAGs through the alignment of query sequences with more than one gene present on NCBI or UCSC databases. Only 232 cases were reviewed and collected in the ConjoinG database from the datasets obtained by the groups of Parra et. al. [4], Akiva et. al. [8] and Kim et al. [26]. The remaining 519 FuTAGs were identified by Prakash group and a sub-set of 353 out 751 FuTAGs were experimentally validated by using RT-PCR and sequencing in different tissues. Ultimately, they have collected a total of 800 different FuTAGs originating from 1542 known parental genes and have sorted them according to the chromosome to which they belong. The database contains different tabs which permit the search of FuTAGs filtering by localization on chromosome, gene symbol, mRNA accession, experimental status and associated disorders. Moreover, it can also align mRNA (or proteins) to sequences collected in the database. Unfortunately, this database is no longer up-to-date [25].

The ChiTaRS database, born in 2012 at Bar-Ilan University, collects about 50,000 transcripts, of which 39,405 are human fusion transcripts verified by RT-PCR, qPCR, RNAseq and mass-spectrometry peptides; the remaining 10,595 transcripts belong to the other seven organisms. ChiTaRS database, in its newest version 3.1 (2017), contains 25 FuTAGs validated in humans, with the latest entry registered in 2014. The web interface of this database displays for each record the link to GeneCard, Uniprot (if the protein is available) and PubMed databases. Furthermore, in the latest version of ChiTaRS, the authors developed a network called chimeric protein–protein interaction (ChiPPI), showing the comparison of the proteins obtained from both single and fusion genes. These chimeric transcripts have been mined by ESTs and mRNAs from GeneBank, ChimericDB, the Database of Chromosomal Rearrangements In Diseases (dbCRID), Translocation breakpoints In Cancer TICdb–TICdb and the Mitelman collection of gene fusions in cancer [27]. The current version 0.9 of the Database of Chromosomal Rearrangements In Diseases (dbCRID), released in 2010, collects 2643 validated human chromosomal rearrangements in the corresponding pathologies. It contains information about the chromosomal breakpoint position, genes involved and junction sequences [28]. Translocation breakpoints In Cancer TICdb–TICdb is a database of translocation events in human cancer, created by University of Navarra in 2007, now at version 3.3, online since August 2013. This database records 1374 fusion sequences of breakpoints, found in human tumors and genes involved [29]. The Mitelman collection, created at the University of Lund, is a database of chromosome aberrations and gene fusions in cancer and at its last update (February 2019) contains 21,477 gene fusions [30].

The Tumor Fusion Gene Data Portal, built in 2015 by the Jackson Laboratory, is a repository of cis- and trans- fusion transcripts from 13 tumor types, using an informatic pipeline for RNA sequencing data analysis from TCGA. The database initially reported 7887 high confidence fusion transcripts [31]. In 2017, Hu et al. increased the number of cancer type at 33, reporting 20,731 fusion transcripts, of which 14,027 are fusion transcripts originating from genes that lie on the same chromosome. Furthermore, 4903 out of 14,027 transcripts are in-frame, thus potentially capable of code for proteins [32].

Recently, Kim and Zhou built the Fusion gene annotation DataBase (FusionGDB), to collect known fusion transcripts from three databases: ChiTaRS 3.1, TumorFusions and TCGA fusions by Gao et al. (2018) [33,34]. Figure 2 shows the history of fusion transcript databases.

### Analysis of FuTAGs in Public Databases

In light of current knowledge, is easy to understand that there is a lack of uniformity of information in these databases about fusion transcripts. Indeed, only a few of them contain records specifically on FuTAGs, e.g., ConjoinG and ChiTaRS 3.1. This is due to the fact that the other databases do not sort according to FuTAGs and sometime not even to inter- or intra-chromosomal rearrangement. In addition, we decided to crosscheck the 25 results from ChiTaRS 3.1 with the ConjoinG database, discovering that the 72% of the entries are present in both the databases (complete dataset is reported in Table S1). 

**Figure 3:**
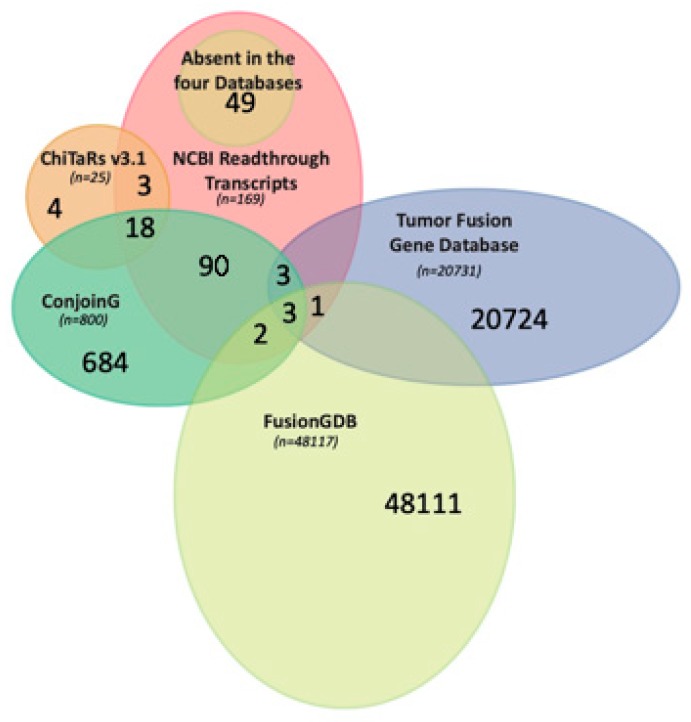
The Venn graph shows the comparison among ChiTaRs v3.1, ConjoinG, the Tumor Fusion Gene Database, Fusion GDB and NCBI readthrough transcripts. The data contained in each dataset are available in Table S2.

The NCBI gene database reports 169 *Homo sapiens* sequences containing the word readthrough in the description (updated May 2019). Furthermore, we have decided to compare these 169 results in the following databases: ChiTaRS 3.1, ConjoinG, Tumor Fusion Data portal and FusionGDB. ChimerDB has not been analyzed, because ChiTaRS 3.1 contains already all of its **FuTAGs**. Comparison of the databases showed that ChiTaRS 3.1 and ConjoinG contain most of the readthroughs deposited into the NCBI, respectively, 21 (12.4%) and 117 (69.23%). Inversely, the Tumor Fusion Gene Data Portal and FusionGDB contain just a few entries, respectively, 7 (0.4%) and 6 (0.3%), underlying their poor usefulness for analysis of FuTAGs. The remaining 49 (28.9%) readthroughs were absent in the aforementioned four databases. The Venn graph (Figure 3) shows how the entries of the aforementioned databases match with the 169 readthrough transcripts reported on NCBI. Only 3 FuTAGs are shared among ConjoinG, FusionGDB and the Tumor Fusion Gene Data Portal. ChiTaRS have 21 FuTAGs matched with NCBI, of which 18 are in common with ConjoinG. In the last one, 116 out of 800 conjoined genes are in common with NCBI, 5 out of 116 are shared with FusionGDB and 3 with the Tumor Fusion Gene Data Portal. Only 1 FuTAG is uniquely shared between FusionGDB and the Tumor Fusion Gene Data Portal. Finally, 49 out of the 169 entries of NCBI are absent in all the other databases.

**Figure 4:**
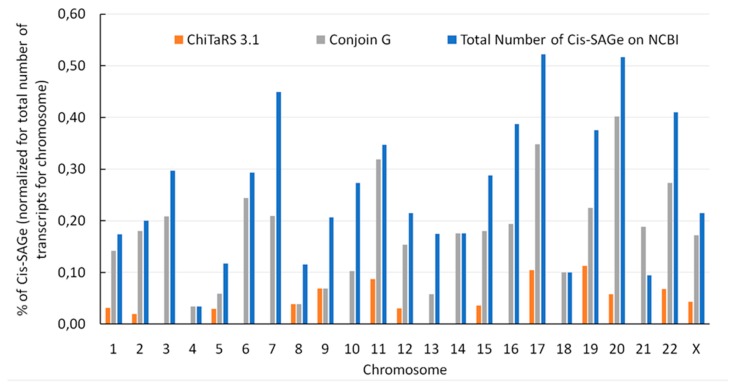
Distribution of FuTAGs in human chromosomes normalized for the total number of transcripts for each chromosome. The results of ChiTaRs v3.1, ConjoinG and NCBI readthroughs are compared.

In addition, we have plotted the distribution of the FuTAGs found on ConjoinG, ChiTaRS and NCBI on human chromosomes. Figure 4 shows the abundance and distribution of the FuTAGs found on ChiTaRS v31, ConjoinG and NCBI on human chromosomes normalized for the total number of transcripts encoded in each chromosome.

## 5. FuTAGs Expression in Normal Tissues and Cancer

In the last years FuTAGs have been revealed in prostate [35], breast [36], ovarian [37] and cervical cancer [11], head and neck squamous cancers [38], bladder urothelial carcinoma [39] and colorectal cancer [40]. Table 1 summarizes the general features of the reported FuTAGs and corresponding parental genes for each one, the chromosomal localization, tissue expression, type of intergenic splicing mechanism (ISP), according to Lu et al.’s classification [10], the NCBI and the Ensembl accession number (NM or NR) for each transcript and structural details about the exons spliced out from the final transcript and, consequently, the exons conjoined in FuTAG. In addition, the junction sequence, the identified from sequence submitted on NCBI or sequencing experiments between two parental genes are reported. The detection and characterization of specific fusion transcripts will increase our knowledge on little explored molecules, such as FuTAGs, in order to identify new candidates useful as biomarkers in the development, progression and prognosis of different subtypes of cancer and to highlight key points needed in the field. Despite several FuTAGs being detected as unique features of tumor cells and tissues, their existence has been also shown in several normal tissues [41], such as prostate cells [42] and normal lung tissues [43]. Examples of FuTAGs involved in cancer are described in detail below.

Magrangeas et al. in 1998 [44] reported the first example of a human FuTAG, GALT-IL11Rα, resulting from intergenic splicing between two adjacent genes. The parental genes, galactose-1-phosphate uridylyl transferase (*GALT* )and interleukin-11-receptor α-chain (*IL-11Rα*), are located on chromosome 9p13. This FuTAG is a cis-SAGe composed of 22 exons as the result of the Type I ISP mechanism, according to Lu et al.’s classification [12], due to an alternative splicing event between the second-to-last exon of the upstream gene and the second exon of the downstream gene (Table 1). The transcription of the GALT-IL11Rα mRNA starts from the upstream gene promoter, which also encodes for *GALT* gene, but the first of two cleavage/polyadenylation signals is detoured to allow cis-SAGe formation (Table 1). cis-SAGe expression was analyzed by RT-PCR, detecting high levels in LT5 cells, LT6 cells and human fetal bone morrow; such results confirmed the presence of GALT-IL11Rα in normal human cells. The transcript encodes for a multiple domains protein placed on the cell membrane, which structure includes a portion of GALT joined to the total amino acid sequence of the IL-11Rα protein. The fusion protein function is unknown and different from parental proteins. Genotype Tissue Expression (GTEx) RNAseq data showed its expression in the following normal tissues: Colon, adipocytes, ovary and testis [45].

Kowalski et al. in 1999 have shown a novel FuTAG expressed in human teratocarcinoma cell lines, known as HHLA1-OC90. The transcript appears to be a fusion between the upstream gene *HHLA1*, whose function is unknown and the downstream gene *OC90* located on chromosome 8q24.22. In physiological conditions the parental genes are transcribed starting from their independent promoters, while FuTAG transcription is induced by human endogenous retrovirus, the long terminal repeat (LTR) promoter, located in an intron. Screening 50 human tissues and cell lines, revealed that only Tera1 and NTera2D1 tetracarcinoma cell lines showed high levels of expression [46].

In 2001, when a mechanism for the readthrough formation was not even known, Communi et al. identified P2Y11-SSF1 (PPAN-P2Y11), located on chromosome 19p13.1, as a co-transcript studied in 11 human tissues. This FuTAG is an example of Type III ISP or the Co-TITE mechanism, since all exons of the upstream gene are joined to the last exon of the downstream gene (Table 1). This transcript codes for a protein and it was the first reported case of a fusion protein involving a G-protein coupled receptor. Its expression has been observed in all tissues, but it seems to be upregulated in HL-60 cells after the induction of granulocyte differentiation. So, Communi et al. defined the formation of this transcript as a common and well-regulated phenomenon [47]. More recently, another group have published conflicting results on the real existence of this fusion transcript and its protein, despite the Genecards [48] for P2Y11-SSF1 reports on both of them [49,50]. The RNAseq data revealed its specific expression in the heart, thyroid, adrenal gland, ovary, prostate and testis [45].

Kato et al. [51] revealed the existence of a FuTAG expressed in Hodgkin and Reed-Sternberg (HRS) cells, related to the progression of Hodgkin’s lymphoma. The cis-SAGe DEC205-DCL1 (or LY75-CD302), located on chromosome 2q24, contains 35 exons from *DEC205* and 6 exons from *DCL1* (Type I ISP; Table 1); it seems that the activation of the readthrough formation is facilitated by the *DEC205* promoter. The parental genes are independently expressed as single genes in hematopoietic cells, but not in HRS cells, where DEC-205-DCL-1 fusion mRNA predominates. Both genes encode for Type I transmembrane lectins, while the cis-SAGe encodes for a fusion protein that contains the DEC-205 ectodomain plus the DCL-1 ectodomain, the transmembrane and the cytoplasmic domain. Kato et al. have hypothesized that the binding between DEC205-ligand and DEC-205/DCL-1 fusion protein could activate a signaling pathway different from that of the DEC205 receptor [51] and suggested the fusion protein as a potential new target for antibody or T cell mediated immunotherapy for Hodgkin’s lymphoma. RNAseq data have assessed its expression in white blood cells, skeletal muscle, thyroid and the adrenal gland [45].

In some prostate cancer cell lines, environmental factors can change the expression level of FuTAG SLC45A3-ELK4, located on chromosome 1q32 and composed by the first exon of *SLC45A3* and the last four of *ELK4* (Type II ISP or Co-TIFE); despite this, the *ELK4* is translated as a wild type protein (Table 1). Zhang et al. [10] discovered high levels of the FuTAG in LNCaP and PC3 prostate cancerous cell lines, while it is absent in normal epithelial prostate cell lines (RWPE-1 and PrEC). This FuTAG regulates proliferation on androgen-dependent and androgen-independent prostate cancer cells. Silencing of SLC45A3-ELK4 transcript inhibits the cell cycle; on the other hand, the downstream gene *ELK4* silencing does not affect the proliferation. The overexpression of this transcript, generally found in metastatic cells, is correlated to a poor prognosis and it could be exploited as a potential biomarker and therapeutic target. FuTAG expression is regulated by the CTCF (CCCTC-Binding Factor) transcription factor, which binds to the insulators located in the proximity of the promoter region of *ELK4*. The more CTCF binds to the insulators, the less the expression of FuTAG. Then, the CTCF ability to bind to insulators between two genes is reduced by androgens treatments, resulting in an enhancing of the expression of this FuTAG [7,10,52,53,54].

Varley et al. [36] identified the following FuTAGs SCNN1A-TNFRSF1A (located on chromosome 12p13.31) and CTSD-IFITM10 (located on chromosome 11p15.5) in breast cancer cell lines, but not in normal tissue. The Type I ISP mechanism generates these two FuTAGs, in agreement with the scheme of Lu et al. (Table 1). Both mRNAs translate into functional proteins because these FuTAGs are in-frame. Silencing of the latter FuTAG produced a decrease in living cells, suggesting its role in breast cancer proliferation. Both fusion proteins, like the normal ones, are located in the membrane, a characteristic that makes them possible candidates as therapeutic targets and/or biomarkers in breast cancer. Despite this, it has been found expressed in normal tissue, compromising the use of this FuTAG as a biomarker [41].

The FuTAG STX16-NPEPL1, located in chromosome 20q13.32, was first identified by Wen et al. [14] in AML (acute myeloid leukemia) and then was validated by Kang et al. [55] in gastrointestinal stromal tumors (GIST) using RT-PCR and Sanger sequencing. The final transcript is obtained by junction of the first seven exons of the upstream gene with the last twelve exons of the downstream gene, splicing out exons eight and nine of STX16 and the first exon of NPEPL1 (Table 1). This FuTAG is recurrent in GIST showing an expression higher than parental genes. This feature underlies the correlation between FuTAG formation and overexpression of genes [55] and buttresses the potential relevance as a marker for clinical application in GIST and AML.

Cheng et al. [38] reported the FuTAG JMJD7-PLA2G4B, located on chromosome 15q15.1 (Table 1), as involved in cell survival, proliferation and cell cycle progression in human head and neck squamous cell carcinoma cell lines. The final transcript is originated from the junction of exon six of the upstream gene and the exon two of the downstream gene (Table 1). The mRNA is translated into a functional protein, containing domains belonging to both genes. It is capable of blocking the cell cycle between the G1 phase and the S phase and it is involved in the phosphorylation of Akt, mediated by the activation of HGF (Hepatocyte Growth Factor), thus acting as oncogene. Downregulation by siRNA reduces the cell proliferation rate; therefore, this FuTAG could be further studied as a potential target for cancer therapy [38]. GTEx RNAseq data showed its ubiquitous expression [45].

Li et al. [56] evaluated the presence of cis-SAGe TSNAX-DISC1, overexpressed in endometrial carcinoma (EC), both in vitro and in vivo. From RNA sequencing of tumoral and corresponding normal tissues, the authors have identified this readthrough transcript, located in chromosome 1q42.2, which comprises the first four exons of TSNAX joined to the last six exons of DISC1. A supplementary exon is added between the two parental genes in the final transcript as a consequence of the Type IV ISP mechanism (Table 1). The expression of this cis-SAGe is regulated by binding of CTCF insulator elements, placed between two parental genes: The binding of CTCF with insulators blocks the cis-SAGe formation. Li et al. have shown an overexpression of lncRNA-NR_034037 in EC, whose sequence is complementary to the intergenic region between the *TSNAX* and *DISC1* genes and competes against CTCF for binding to insulator elements. Thus, the binding of lncRNA-NR_034037 to the insulators is directly correlated to cis-SAGe expression by promoting G1-S cell cycle progression and tumor development. The authors indicate that the expression of TSNAX-DISC1 regulated by lincRNA-NR_034037 could have a key role in the progression of EC and suggest it as a potential new genetic marker in EC [56]. GTEx RNAseq data showed its ubiquitous expression [45].

From the analysis of the stomach adenocarcinoma RNAseq dataset, Choi et al. [57] selected three possible FuTAG candidates involved in gastric cancer: PHOSPHO2-KLHL23 (Type I ISP), RPL17-C18orf32 (Type I ISP) and PRR5-ARHGAP8 (N.D. ISP; (Table 1). Initially, these transcripts were validated by RT-PCR in gastric cancer cell lines and then their expression was evaluated in tumor tissues compared with mucosae. All candidates have greater expression in tumor tissues than normal samples, but only the FuTAG PHOSPHO2-KLHL23 showed a correlation with clinicopathological features of gastric cancer. The parental genes constituting the FuTAG, PHOSPHO2 and KLHL23 (chromosome 2q31.1) are involved in the cell growth. The readthrough transcript PHOSPHO2-KLHL23 is translated into the downstream gene protein KLHL23. In order to evaluate the involvement of this FuTAG in the tumor progression, the construct PHOSPHO2-KLHL23 was transfected into HEK-293 cells showing the correlation between its expression and perineural invasion in gastric cancer. Since promoter methylation could be involved in cis-SAGe formation, the authors revealed a low methylation of PHOSPHO2-KLHL23 promoter. Thus, considerable methylation of the KLHL23 promoter inhibits its transcription and promotes the readthrough formation [57].

The *MASK* and *EIF4EBP3* genes, located on chromosome 5q31.3, are the components of the FuTAG MASK-BP3, also called ANKHD1-EIF4EBP3, which comprises thirty-three exons belonging to *MASK* and three exons belonging to *4E-BP3*, separated by an intermediate exon (composed by 110 bp) resulting from the Type IV ISP mechanism (Table 1). So the FuTAG could be the result of a Type V ISP according to Lu et al.’s classification [12]. The authors hypothesized that the formation of this cis-SAGe is due to a weak termination signal in the upstream gene *MASK* and the result is a readthrough transcription of the downstream gene. The two proteins, MASK and 4E-BP3, are separately translated from the FuTAG MASK-BP3, using alternative reading frames for the downstream gene *4E-BP3* in the second exon. In this way, since no premature stop codon is observed in MASK-BP3 transcript, the nonsense-mediated decay mechanism cannot be activated. The proteins are involved in the same biochemical pathway: MASK activates the Ras/MAPK signal pathway, regulating the phosphorylation of 4E-BP3 and its interaction with eIF4E subunit of eIF4F (initiation factor 4F) involved in the control of translation rate. This underlies the possible role of this FuTAG as an oncogenic factor [58]. GTEx RNAseq data show its ubiquitous expression in normal human tissues [45].

Grosso et al. [13], analyzing a TCGA dataset of 50 matched samples of clear cell renal cell carcinoma (ccRCC), noticed a frequent formation of FuTAG. The study was aimed to find a correlation between the most mutated genes in ccRCC and readthrough formation. *SETD2* was inversely correlated to FuTAG expression: Mutations on this gene resulted in an increase of readthrough events. Moreover, they identified the FuTAG CTSC-RAB38, located on chromosome 11q14.2, expressed in 20% of the TCGA samples. Experimental silencing of the last exon of *CTSC* and of the first of *RAB38* (exons not present in the readthrough mRNA) in ccRCC cell lines resulted in a downregulation of single gene mRNA, but not the FuTAG [13].

Wu et al. [11] compared the expression of FuTAGs on cervical cancer tissue, PAP smear (Papanicolaou test) and normal epithelia, identifying SLC2A11-MIF to be significantly more expressed in the cancer than the normal epithelia. This FuTAG includes the first eight exons (twelve exons in total) of the upstream gene joined to the second exon and the third (three exons in total) of the downstream gene as a result of modified Type I ISP, according to Lu et al.’s classification (Table 1) [12]. A silencing experiment of this FuTAG transcript showed a significant arrest in the cell cycle, demonstrating its involvement in CDKN1A pathways [11]. 

Recently, Gao et al. [59] discovered a new FuTAG, INS-IGF2, originating from the *INS* and *IGF2* genes, located on chromosome 11q15.5, that acts as a lncRNA. It has been observed upregulated in NSCLC (Non-Small Cell Lung Cancer) tissue, but not in the adjacent tissue (Table 1). 

The sequence on NCBI reveals that the final transcript originated from the second-to-last exon of the upstream gene and the first exon of the downstream gene, splicing out the third and a part of the first exon of INS and IGF2, respectively (Table 1). Downregulation by a siRNA against the FuTAG produced a decreased expression of the single gene *IGF2*, blocking cells between G1/S phases. This gene codes for Insulin Growth Factor 2, a peptide hormone involved in cell growth, differentiation and metabolism. The upregulation of this FuTAG is considered oncogenic and the authors speculate the possible use in therapy for NSCLC patients [59]. GTEx RNAseq data show its ubiquitous expression in normal human tissue [45].

The FuTAG NFATC3-PLA2G15, is composed by the first nine exons of the upstream gene and the exons from two to six of *PLA2G15* (Type I ISP), both located on chromosome 16q22.1 (Table 1). Wen et al. [14] identified, as previously reported, this FuTAG by pair-end RNAseq analysis on acute myeloid leukemia (AML) samples. Validation of NFATC3-PLA2G15 showed the presence of a valine in the fusion area between two parental genes. This amino acid is encoded by the following codons in single transcripts: GTG and GTC, located in the junction between exon 9 and 10 of NFATC3 and in the junction between exon 1 and 2 of PLA2G15, respectively. So, the final protein sequence of FuTAG comprised one valine, which is encoded by GTC codon (G from the upstream gene and TC from the downstream gene) [14]. This FuTAG is generally upregulated in T-acute lymphoblastic leukemia, but not in healthy tissues. Increase in expression of NFATC3-PLA2G15 is correlated to a bad prognosis and usually with a more rapid leukemia development [60]. Moreover, the expression of this FuTAG has been confirmed in colorectal cancer, where it is correlated with the epithelial-mesenchymal transition, as confirmed by silencing assay [40].

This review of scientific literature on FuTAGs highlights the large functional heterogeneity of this class of molecules. They could act forming novel fusion proteins bearing new functional properties or act as long non-coding RNA involved in both structural and functional activities or they could represent a novel mechanism for regulation of parental gene expression.

## 6. FuTAG’s Parent Genes: RNAseq and Transcriptome Microarray (HTA 2.0) Data

**Figure 5:**
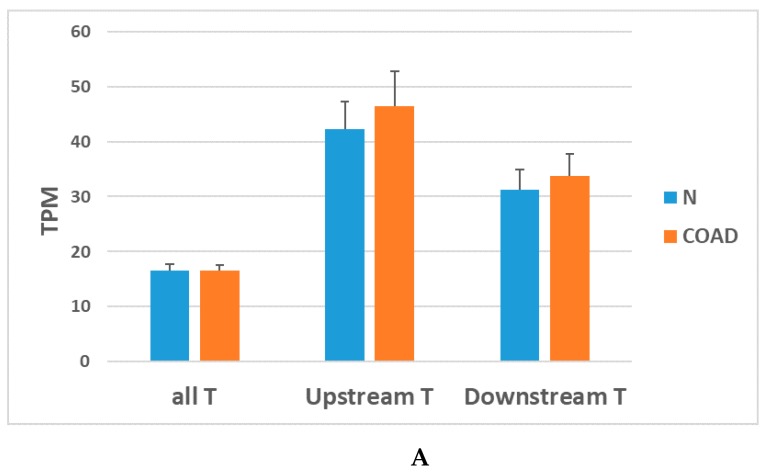

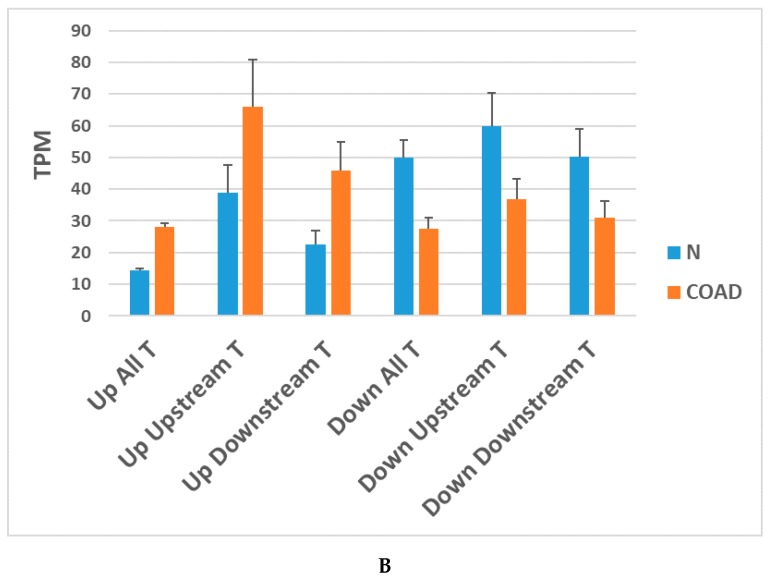
(**A**) Averages (±SEM) of TPM values of all transcripts (all T), upstream ConjoinG transcripts (Upstream T), including both the upstream parent gene and the upstream part of the fusion transcript and downstream ConjoinG transcripts (Downstream T), including both the downstream parent gene and the downstream part of the fusion transcript. N: Normal colonic mucosae; COAD: CIN-positive colon adenocarcinomas from TCGA. (**B**) Averages (±SEM) of TPM values of all 60,485 analysed transcripts (All T), Upstream transcripts (Upstream T) and Downstream transcripts (Downstream T). Up: Upregulated; Down: Downregulated.

Analysis of FuTAGs expression separately from that of their parent genes requires techniques based on hybridization, sequencing or amplification, that exploit the presence of novel intergenic exons or specific splice junctions. For instance, RNA sequencing (RNAseq) data should be analysed with specific algorithms able to differentiate between parent transcripts and fusion ones. However, taking into account that the expression values of parent transcripts are contaminated by those of FuTAGs, some interesting information can be obtained by analysing the large amount of processed RNAseq data publicly available from The Cancer Genome Atlas (TCGA) consortium. Using RNAseq data of 123 colon adenocarcinoma (COAD) samples with chromosomal instability (CIN positive) and 42 samples of normal colonic tissue we calculated the average transcript level, expressed as transcripts per million (TPM), of 800 transcripts reported in the database ConjoinG. As shown in Figure 5A the average TPM value of ConjoinG upstream transcripts (including both the upstream parent gene and the upstream part of the FuTAG) is higher than the average value of all 60,485 transcripts analysed by RNAseq. Moreover, upstream transcripts show higher TPM values than the downstream ones, although such difference is not statistically significant. These data suggest that genes involved in FuTAG formation are among highly expressed genes and that a trend towards a higher expression of the upstream gene in comparison to the downstream gene can be observed. No significant difference between tumor and normal colon tissue is detectable comparing average TPM values. 

However, genes involved in FuTAG formation are differentially expressed between tumor and normal tissues. Indeed, about 35–39% of genes involved in FuTAG (upstream or downstream) are upregulated (fold-change, FC, between COAD and normal tissue >1 at a false discovery rate (FDR) *p* value < 0.05) and 32–34% are downregulated (FC < 1 at FDR < 0.05) [64]. For comparison it can be noted that only 23% of all analysed transcripts are upregulated and 17% downregulated.

In Figure 5B average TPM values are reported separately for upregulated (Up) or downregulated (Down) transcripts, showing higher TPM values for upregulated upstream transcripts in comparison to downstream ones.

**Figure 6:**
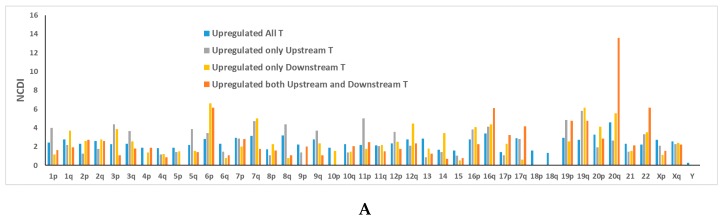

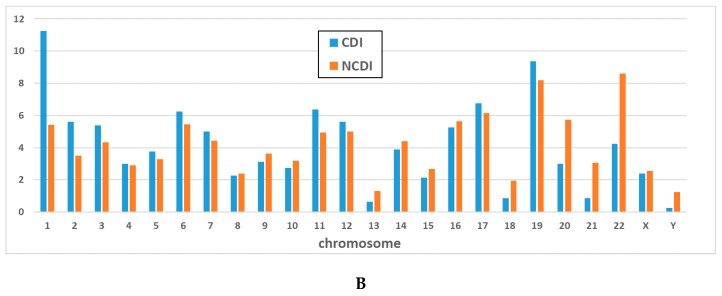
(**A**) Percentage chromosomal distribution of upregulated FuTAG’s parent genes normalized for the total number of transcripts in each chromosome (normalized chromosomal distribution index (NCDI)). (**B**) Percentage chromosomal distribution of 800 ConjoinG transcripts (Chromosomal distribution index (CDI)) and its normalized chromosomal distribution index, NCDI (CDI normalized for the total number of transcripts in each chromosome).

These data allowed us to calculate which proportion of upregulated FuTAGs shows a significant increase of only the upstream transcript (40%), only the downstream transcript (36%) or both the upstream and downstream transcripts (24%). Moreover, Figure 6A shows the percentage of chromosomal distribution of upregulated FuTAG’s parent genes normalized for the total number of transcripts in each chromosome (normalized chromosomal distribution index (NCDI)). Interestingly, a high density of upregulated FuTAG’s parent genes, showing a simultaneous upregulation in both the upstream and downstream genes, can be observed in chromosome 20q. This is not a simple reflection of a high density of FuTAGs (800 ConjoinG transcripts) in chromosome (Chr) 20 (Figure 6B), since the highest NCDI values of ConjoinG FuTAGs are observed in Chr19 and 22. 

Chr20q is the chromosome most frequently affected by arm-level copy number aberrations of the gain-type (such as trisomy and tetrasomy) [65,66].

Table 2 shows readthrough transcripts located in Chr20q and upregulated in comparison with normal mucosa in both parent genes. Counts relative to some readthrough transcripts (101 transcripts) are also provide in processed RNAseq data from TCGA and are reported in one of the columns of Table 3 if available for ConjoinG transcripts. However, as already pointed out previously, the method used to derive such values does not distinguish between parent gene transcripts and FuTAG transcripts and does not provide a specific quantification of readthrough transcript levels. Specific methods should be applied in further study to evaluate the quantitative relationship between parent transcripts and corresponding FuTAGs. Interestingly, Thomson et al (2000) have already shown, several years ago, that one of the readthrough transcripts reported in Table 3, the Kua-UBE2V1, is expressed as a hybrid transcript and protein in several cancer cell lines, including colon cancer cells (see Section 4 for further functional data). According to an estimate of these authors, based on PCR amplification results, the ratio of *Kua–UBE2V1* to *Kua* ranged from 0.1 to 0.02.

Furthermore, we evaluated the possibility to assess the readthrough transcripts using a Human Transcriptome Array 2.0 chip (HTA) (Affymetrix, Santa Clara, CA, USA), able to analyze over 67,000 transcripts, both coding and non-coding [66,67,68]. Starting from a Transcription Analysis Console (TAC) dataset (Affymetrix, USA), we filtered for readthrough and found 95 Transcript Clusters (TCs) potentially identifiable with HTA analysis. We have considered only 81 out of 95 results, due to the presence of redundancy (Table S2). Then, we paired these 81 TCs, obtained by analysis of HTA chip, with 169 readthrough transcripts included in NCBI datasets (data available in Table S2). The result of this comparison shows that 3 out of 81 are absent in NCBI. Of course, analysis of TCs in HTA does not provide a specific estimate of readthrough transcripts. Indeed, the majority of probes contained in those TCs are directed against sequences present also in the parent genes (upstream or downstream genes) as reported in Table 3. In some cases, TCs contain also probes against small nucleolar RNAs embedded in the parent genes of FuTAGs and this inclusion causes a strong bias in the result. Therefore, results obtained by HTA analysis can only provide a rough estimate of the expression of parent genes of FuTAGs. 

The transcriptome analysis performed by HTA in colorectal cancer samples (data deposited to public repository Gene Expression Omnibus (GEO) (www.ncbi.nlm.nih.gov/geo) and accessible through GEO: GSE73360 and GSE84984) [66,67,68] confirmed the upregulation of the parent genes of FuTAGs located in Chr20 observed by RNAseq analysis (data reported in Table 3). Moreover, this analysis revealed that, among 78 readthrough transcripts, 20 readthrough transcripts are significantly increased with a fold change > 1.5 in comparison to normal tissue (FDR < 0.05) (FC is the linear fold change obtained comparing all CRC samples with matched normal colonic mucosae as previously described in Condorelli et al., [66] (Table 3). Among these four readthrough transcripts, PHOSPHO2-KLHL23 [57], LY75-CD302 [51], ANKHD1-EIF4EBP3 [58] and TMED7-TICAM2 [69] matched with those previously reported in the literature (Table 1 and Table 3).

## 7. Downstream of Gene Containing Transcripts and cis-SAGes

Downstream of gene containing transcripts (DoGs) recently described by Steitz’s research group [21,70] are very long transcripts generated by readthrough transcription of upstream protein-coding genes. Vilborg et al. [21] have shown that heat shock, osmotic stress and oxidative stress increase transcriptional readthrough and DoGs formation. Moreover, transcriptional readthrough is differentially induced across different stress conditions. The authors demonstrated by two separate experiments, using in the first Actinomycin D to inhibit the transcription and, in the second the analog 5-ethynyl uridine to label newly synthesized transcripts, that, in some circumstances as in the osmotic stress, RNA polymerase II engages in a productive elongation of the upstream gene continuing through the transcription termination site (TTS) and transcribing the downstream gene in order to produce a primary RNA containing both transcripts (upstream and downstream). In this case, they observed a reduced transcription termination of the upstream gene. DoGs were revealed using a combination of two procedures: RNAseq of total RNA (RNAseq) and analysis of capped sequence (Cap-Seq) as reported by Xie et al. [71]. The authors revealed that DoGs possess long non-coding regions (often >45 kb) that remain chromatin bound and that they are inducible by osmotic stress through an IP3 (Inositol 1,4,5-Trisphosphate) receptor signaling-dependent pathway. They detect DoG transcription in several human cell lines and provide evidence for thousands of DoGs genome-wide. DoG-associated genes show a significant enrichment of histone marks typical of open chromatin state (H3K4me1 and H3K27ac) and elongation histone marks (H3K36me3 and H3K79me2), but no significant difference for the repressive mark H3K27me3. Moreover, analysis of publicly available datasets obtained by the DNase hypersensitivity technique (DNase-seq) in NIH 3T3 cells and by Assay for transposase-accessible chromatin using sequencing (ATAC-seq) in mouse embryonic fibroblast cells (MEF cells) revealed a significant enrichment in active chromatin sites (DNase hypersensitive sites and ATAC-seq peaks), both in the promoters and downstream of pan-stress DoG genes [21,70]. It has been suggested that the DoGs retention at their sites of transcription maintains the euchromatin state and reinforces the nuclear scaffold in response to osmotic and/or heat stresses.

Chwalenia et al. [20] addressed the questions whether cis-SAGes are also induced under osmotic stress and whether the DoGs are correlated to the formation of cis-SAGes. They studied five cis-SAGe RNAs (CTNNBIP1-CLSTN1, DUS4L-BCAP29, CLN6-CALML, SLC29A1-HSP90AB1, UBA2-WTIP) that have DoGs from their upstream parental genes and evaluated their expression in experimental conditions of osmotic stress. Only at a late time after osmotic stress (24 h time point) were cis-SAGe RNAs and the corresponding DoGs positively correlated and upregulated by osmotic stress. Chwalenia et al (2017) [20] suggested that osmotic stress induces more transcriptional readthrough, with some transcripts remaining as DoGs and some processed into cis-SAGes. However, the relationship between DoGs and cis-SAGes is not clear and the functional connections between these two different phenomena require more investigations.

This year, Chwalenia et al. [72] tuned an assay to investigate the fusion transcript formation and its regulation. The authors have designed an assay consisting of two detectors (Renilla and Firefly luciferase) in order to assess the actors involved in the expression of readthrough CTNNBIP1-CLSTN1. Once the cis-SAGe is spliced, the Renilla is expressed too and the ratio between Renilla and Luciferase intensities is used to assess the cis-SAGe formation. Chwalenia et al. have selected some trans-acting regulators involved in the RNA polymerase cleavage and termination, elongation, splicing and R-loop formation according to the DLR assay. The activity of *SF3B1* and *SRRM1* was tested on five cis-SAGes by silencing experiment and the results suggested that *SRRM1* acts as a negative regulator of readthrough expression, while *SF3B1* acts as a positive regulator of cis-SAGe formation [72].

## 8. Conclusions

Since their discovery, FuTAGs were considered as cancer-signature transcripts, but some studies suggest that they exist in physiological cells too, discrediting them as unique among cancer cells [73]. Some FuTAGs, found in multiple tissue and cell types, have been suggested to play some basic cellular maintenance roles.

It has been estimated that the readthrough phenomenon occurs in the 4–6% of human genomes [4,8,74]. Some authors have reported the possibility of using some FuTAGs as biomarkers for therapeutic response assessment and non-invasive diagnosis [10,36,52,53,54,75], but the discovery of readthrough transcripts and proteins in physiological cells and tissues could belie the effectiveness of the use in diagnostics [41,42,43,61]. Since the low statistical power of the majority of these studies do not allow clear and sound responses, more robust and reliable data are necessary to assess the real role of FuTAGs and the development of clinical applications for these types of fusion RNA.

Our analysis of public databases brought to light a lack of uniformity and specificity for FuTAGs. Furthermore, some databases are the elaboration of data acquired by other databases, which data are re-elaborated and integrated to newer datasets creating a Chinese-box mechanism. Thus, it is really complicated to track the origin of the data reported in these databases. Only two of these, namely ChiTaRs v.3.1 and ConjoinG, showed the most matches with the NCBI reported readthrough and they have even shown to have matches between themselves. Unfortunately, with its 800 readthroughs, ConjoinG has not been updated since its release in 2010; nevertheless, it is the most complete public database for FuTAGs. The use of massive and parallel techniques, like NGS and arrays, could be the answer to more robust studies. 

In conclusion, further research is necessary to assess the real role of each FuTAG in pathological and physiological conditions and more data on the mechanisms that govern the expression of the readthrough transcripts have to be discovered.

## Figures and Tables

**Table 1:** List of experimentally evaluated FuTAGs. Additional exons are highlighted in green letters. Chr: Chromosome; NM and NR: NCBI curated Refseq accession numbers for coding and non-coding transcripts, respectively.

N.	FuTAG	Upstream Gene	Downstream Gene	Position (Chr)	Tissue/Cell Type	Normal Tissue Expression (GTEx)	NM, NR	ISP Mechanism in According to Lu et al., [12]	Ensembl Code	Structure	Junction Exon Sequence	Reference
1	GALT-IL11Rα	*GALT*	*IL11Rα*	9p13	Normal human cell- T cell clones and fetal bone marrow	Colon, adipocytes, ovary and testis	N.D.	Type I	ENSG00000258728	ex^10^-ex^2^ (ex^11^-ex^1^ removed)	GAGCAG-ATGAGC	Magrangeas et al., [44]
2	HHLA1-OC90	*HHLA1*	*OC-90*	8q24.1–24.3	Tera1 and NTera2D1 cell lines	N.D.	N.D.	N.D.	N.D.	N.D.	N.D.	Kowalski et al., [46]
3	P2Y11 (PPAN)-SSF1	*P2Y11*	*SSF1 (PPAN)*	19p13.1	HL-60 cell lines	Heart, thyroid, adrenal gland, ovary, prostate and testis	NM_001040664; NM_001198690	Type III	ENSG00000243207	ex^12partial^-ex^2^ (ex^12partial^-ex^1^ removed)	ATCGAG-GTGCCA	Communi et al., [47]
4	TWE-PRIL (TNFSF12-TNFSF13)	*TWEAK (TNFSF12)*	*APRIL (TNFSF13)*	17P13.1	T lymphocytes and monocytes cell lines	Kidney, liver and breast	NM_172089	Type I	ENSG00000248871	ex^6^-ex^2^ (ex^7^-ex^1^ removed)	TGTCAG-AGTTCC	Pradet-Balade et al., [16]
5	SLC45A3-ELK4	*SLC45A3*	*ELK4*	1q32	LNCaP and PC3 prostate cancer cell lines	N.D.	N.D.	Type II	N.D.	N.D	N.D	Kumar et al., [52]
6	DEC205-DCL1 (or LY75-CD302)	*DEC205 (LY75)*	*DCL1 (CD302)*	2q24	Hodgkin and Reed-Sternberg cells	White blood cells, skeletal muscle, thyroid, adrenal gland	NM_001198759	Type I	ENSG00000248672	ex^34^-ex^2^ (ex^35^-ex^1^ removed)	CTCTGG-ACTGTC	Kato et al., [51]
7	SCNN1A-TNFRSF1A	*SCNN1A*	*TNFRSF1A*	12p13.31	Breast cancer cell lines	N.D.	N.D.	Type I	N.D.	ex^12^-ex^2^ (ex^13^-ex^1^ removed)	GTCACG-GTGCTC	Varley et al., [36]
8	CTSD-IFITM10	*CTSD*	*IFITM10*	11p15.5	Breast cancer cell lines	N.D.	N.D.	Type I	N.D.	ex^8^-ex^2^ (ex^9^-ex^1^ removed)	CTCAAG-GCCCAG	Varley et al., [36]
9	STX16-NPEPL1	*STX16*	*NPEPL1*	20q13.32	Acute myeloid leukemia and gastrointestinal stromal tumors	Whole blood, lymph node, brain, cortex, cerebellum, spinal cord, heart, artery, skeletal muscle, small intestine, colon, adipocyte, kidney, liver, lung, spleen, stomach, esophagus, bladder, pancreas, thyroid, salivary gland, adrenal gland, pituitary, breast, skin, ovary, uterus, placenta, prostate, testis.	NR_037945.1	Type IV	ENSG00000254995	ex^8^- ex^1(addictional intergenic exon)^- ex^2(addictional intergenic exon)-^ ex^3(addictional intergenic exon)^-ex^2-6^- ex^1(addictional intron exon)^ -ex^7-12^ (ex^9^-ex^1^ removed)	CACAAG-GACTTC_CACACT-TGCCTG_GGGAAG-GCTGGT_ATGGAG-CTCTGG_GGGAAG-AGGGCA_GGGGGT-ACTACC	Wen et al. [14]; Kang et al. [55]
10	JMJD7-PLA2G4B	*JMJD7*	*PLA2G4B*	15q15.1	human head and neck squamous cell carcinoma cell lines	White blood cells, lymph node, brain, heart, colon, adipocyte, kidney, liver, lung, thyroid, adrenal gland, breast, ovary, prostate, testis.	NM_001198588; NM_005090	N.D.	ENSG00000168970	ex^6^-ex^2^ (ex^7^-ex^1^ removed)	GAGAAG-GCAGAG	Cheng et al., [38]
11	miR-200c/141-PTPN6	*miR-200c/141*	*PTPN6*	N.D.	Ovarian tumorigenesis	N.D.	N.D.	N.D.	N.D.	N.D	N.D	Batista et al., [37]
12	DUS4L-BCAP29	*DUS4L*	*BCAP29*	7q22.3	gastric and prostate cancer tissues	N.D.	N.D.	Type I	N.D.	ex^7^-ex^2^ (ex^8^-ex^1^ removed)	CAGATG-GTGTGA	Tang et al., [61]
13	TSNAX-DISC1	*TSNAX*	*DISC1*	1q42.2	endometrial carcinoma tissues	Whole blood, brain, cortex, cerebellum, spinal cord, tibial nerve, heart, artery, skeletal muscle, small intestine, colon, adipocyte, kidney, liver, lung, spleen, stomach, esophagus, bladder, pancreas, thyroid, salivary gland, adrenal gland, pituitary, breast, skin, ovary, uterus, prostate, testis.	NR_028393; NR_028394; NR_028395; NR_028396; NR_028397; NR_028398; NR_028399; NR_028400	Type IV	ENSG00000270106	ex^4^-ex^(addictional intergenic exon)^-ex^2^ (ex^5/6^-ex^1^ removed)	ACTACA-AAGTTT_TATTTG-GCAGCC	Li et al., [56]
14	PHOSPHO2-KLHL23	*PHOSPHO2*	*KLHL23*	2q31.1	Gastric cancer cell lines and tissues	N.D.	NM_001199290; NR_144936	Type I	ENSG00000213160	ex^3^-ex^2^ (ex^4^-ex^1^ removed)	AGTTGG-CCATGG	Choi et al., [57]
15	RPL17-C18orf32	*RPL17*	*C18orf32*	18q21.1	Gastric cancer cell lines and tissues	N.D.	NM_001199355; NM_001199356	Type I	ENSG00000215472	ex^6^-ex^2^ (ex^7^-ex^1^ removed)	AAAAAG-TTGAGG	Choi et al., [57]
16	PRR5-ARHGAP8	*PRR5*	*ARHGAP8*	22q13.31	Gastric cancer cell lines and tissues and bipolar disorder	White blood cells, brain, colon, adipocyte, kidney, lung, thyroid, adrenal gland, breast, ovary, prostate, testis.	NM_181334	N.D.	ENSG00000248405	ex^4^-ex^2^ (ex^5–8^-ex^1^ removed)	ATGAGG-AGCTGC	Choi et al., [57]; McElroy et al., [62]
17	Kua-UVE1 (TMEM189-UBE2V1)	*Kua*	*UVE1*	20q13.2	Colon cancer cell lines	Liver, thyroid, adrenal gland, breast, testis.	NM_199203	Type I	ENSG00000124208	ex^5^-ex^2^ (ex^6^-ex^1^ removed)	CCACAG-GAGTAA	Thomson et al., [17]
18	MASK-BP3 (ANKHD1-EIFAEBP3)	*MASK*	*EIF4EBP3*	5q31.3	?	White blood cells, lymph node, brain, heart, skeletal muscle, colon, adipocyte, kidney, liver, lung thyroid, adrenal gland, breast, ovary, prostate testis.	NM_020690	Type IV	ENSG00000254996	ex^33^-ex^(addictional intergenic exon)^-ex^2^ (ex^34^-ex^1^ removed)	CAGCAG-GCCAGT_CCAGAG-GCACCA	Poulin et al., [58]
19	CTSC-RAB38	*CTSC*	*RAB38*	11q14.2	Clear renal cell carcinoma	N.D.	N.D.	N.D.	N.D.	N.D	N.D	Grosso et al., [13]
20	BC039389-GATM (WRB-SH3BGR or KLK4-KRSP1 )	*WRB*	*SH3BGR*	21q22.2	Kidney cancer	N.D.	NM_001317744; NM_001350300	N.D.	ENSG00000285815	N.D	N.D	Pflueger et al., [63]
21	LHX6-NDUFA8	*LHX6*	*NDUFA8*	N.D.	Cervical cancer tissues (PAP smear)	N.D.	N.D.	N.D.	N.D.	Variant.1- ex^8^-ex^2^ (ex^9–10^-ex^1^ removed) Variant.2- ex^8^-ex^3^ (ex^9/10^-ex^1/2^ removed)	ACTTGA-GTGAAA ACTTGA-GCAGAT	Wu et al., [11]
22	SLC2A11-MIF	*SLC2A11*	*MIF*	N.D.	Cervical cancer tissues (PAP smear)	N.D.	N.D.	N.D.	N.D.	ex^9^-ex^2^ (ex^10–13^-ex^1^ removed)	GTTAGT-TACATC	Wu et al., [11]
23	INS-IGF2	*INS*	*IGF2*	11q15.5	NSCLC tissues	Whole blood, brain, cortex, cerebellum, spinal cord, tibial nerve, heart, artery, skeletal muscle, colon, adipocyte, kidney, liver, lung, stomach, esophagus, pancreas, thyroid, salivary gland, adrenal gland, pituitary, breast, ovary, testis.	NM_001042376; NR_003512	N.D.	ENSG00000129965	ex^2^-ex^1partial^ (ex^3^-ex^1partial^ removed)	TGCAGG-CCTCAG	Gao et al., [34]
24	NFATC3-PLA2G15	*NFATC3*	*PLA2G15*	16q22.1	T-acute lymphoblastic leukemia and Colon rectal cancer	N.D.	N.D.	Type I	N.D.	ex^9^-ex^2^ (ex^10^-ex^1^ removed)	ATGATG-TCCCTG	Bond et al., [60]; Jang et al., [40]
25	BCL2L2-PABPN1	*BCL2L2*	*PABPN1*	14q11.2	Bladder urothelial carcinoma tissues and cell line.	Whole blood, brain, cortex, cerebellum, spinal cord, tibial nerve, heart, artery, skeletal muscle, small intestine, colon, adipocyte, kidney, liver, lung, spleen, stomach, esophagus, bladder, pancreas, thyroid, salivary gland, adrenal gland, pituitary, breast, skin, ovary, uterus, prostate, testis.	NM_001199864	Type I	ENSG00000258643	ex^3^-ex^2^ (ex^4^-ex^1^ removed)	GGCTGG-GAGCTG	Zhu et al., [39]
26	CHFR-GOLGA3	*CHFR*	*GOLGA3*	12q24.33	Bladder urothelial carcinoma tissues and cell line.	N.D.	N.D.	Type I	N.D.	N.D	N.D	Zhu et al., [39]

**Table 2:** FuTAGs located in Chr20q and upregulated in comparison with normal mucosa in both parent genes. Data obtained by RNAseq have been explored in HTA 2.0.

Conjoined Genes (ConjoinG ID and Name)	Omics Technologies	Alias	Upstream Gene	Downstream Gene	Readthrough	RNA*	Known Hybrid Protein*	Chr	Band
			FC **	FC **	FC **				
CGHSA0796 NFS1-CPNE1	RNAseq		1.58	3.36	N/A	NO	NO	20	q11.22
	HTA2.0		4.52	1.53	4.52				
CGHSA0023 TGIF2-C20orf24	RNAseq		N/A	N/A	2.08	YES	CDS Predicted	20	q11.23
	HTA2.0		1.11	2.13	N/A				
CGHSA0579 TP53RK-SLC13A3	RNAseq		2.39	20.324	N/A	YES	NO	20	q13.12
	HTA2.0		1.29	−1.25	N/A				
CGHSA0573 SPINLW1-WFDC6	RNAseq	EPPIN-WFDC6	7.69	2.97	1.45	NO	NO	20	q13.12
	HTA2.0		−1.69	−1.9	−1.69				
CGHSA0217 Kua-UBE2V1	RNAseq	TMEM189-UBE2V1	1.59	1.42	1.24	YES	YES	20	q13.13
	HTA2.0		1.72	1.41	1.72				
CGHSA0215 STX16-NPEPL1	RNAseq		2.07	2.54	3.05	YES	YES	20	q13.32
	HTA2.0		1.85	1.1	1.51				
CGHSA0738 SLMO2-ATP5E	RNAseq	PRELID3B-ATP5F1E	1.36	1.33	N/A	YES	NO	20	q13.32
	HTA2.0		3.13	2.79	7.11				
CGHSA0212 ZGPAT-LIME1	RNAseq		1.42	3.08	N/A	YES	CDS Predicted	20	q13.33
	HTA2.0		1.1	−1.08	N/A				
CGHSA0570 LIME1-SLC2A4RG	RNAseq		3.08	1.32	N/A	NO	NO	20	q13.33
	HTA2.0		−1.08	1.03	N/A				
CGHSA0214 MYT1-PCMTD2	RNAseq		2.22	2.23	N/A	YES	YES	20	q13.33
	HTA2.0		−1.57	2.7	N/A				
CGHSA0577 TPD52L2-DNAJC5	RNAseq		1.93	1.07	N/A	Not Attempted Experimentally	NO	20	q13.33
	HTA2.0		2.32	1.09	N/A				

* experimentally confirmed by Akiva et al., [8]. ** FC: Linear fold-change in the comparison tumor vs. normal tissue (only transcripts showing an FC value > 1.5 in one of the two parent genes in RNAseq data are reported). N/A: Not Available.

**Table 3:** FuTAGs located in all chromosomes and upregulated (FC > 1.5) in CRC in comparison to normal mucosa. Data obtained by HTA 2.0.

Transcript Cluster ID	FC > 1.5 (CRCvs.MU) GSE73360 and GSE84984 [66,67,68]	FDR *p*-Value (CRC VS. MU)	Chr Position	Gene Symbol	Description (Contain Readthrough Word)	FuTAG Reported in Table 1
TC02005002.hg.1	1.57	2 × 10^−6^	2q31.1	KLHL23; PHOSPHO2-KLHL23	kelch-like family member 23; PHOSPHO2-KLHL23 readthrough; NULL	[57]
TC02005005.hg.1	2	1.7 × 10^−7^	2q33.1	MOB4; HSPE1-MOB4	MOB family member 4, phocein; HSPE1-MOB4 readthrough; NULL	
TC02002467.hg.1	2.32	2 × 10^−6^	2q24.2	LY75-CD302; CD302; LY75	LY75-CD302 readthrough; CD302 molecule; lymphocyte antigen 75; NULL	[51]
TC05000726.hg.1	2.61	1.2 × 10^−7^	5q31.3	EIF4EBP3; ANKHD1; ANKHD1-EIF4EBP3	eukaryotic translation initiation factor 4E binding protein 3; ankyrin repeat and KH domain containing 1; ANKHD1-EIF4EBP3 readthrough; NULL	[58]
TC05001690.hg.1	1.67	2 × 10^−6^	5q22.3	TMED7-TICAM2; TICAM2; TMED7	TMED7-TICAM2 readthrough; toll-like receptor adaptor molecule 2; transmembrane emp24 protein transport domain containing 7; NULL	[69]
TC07003311.hg.1	1.75	1.4 × 10^−5^	7q11.23	DTX2P1-UPK3BP1-PMS2P11; LOC100132832	DTX2P1-UPK3BP1-PMS2P11 readthrough transcribed pseudogene; PMS2 postmeiotic segregation increased 2 (S. cerevisiae) pseudogene	
TC0X002317.hg.1	1.64	1 × 10^−10^	Xq22.1	RPL36A; RPL36A-HNRNPH2	ribosomal protein L36a; RPL36A-HNRNPH2 readthrough; NULL	
TC0X002316.hg.1	4.2	4.1 × 10^−12^	Xq22.1	HNRNPH2; RPL36A-HNRNPH2	heterogeneous nuclear ribonucleoprotein H2 (H’); RPL36A-HNRNPH2 readthrough; NULL	
TC10002935.hg.1	2.17	5.4 × 10^−9^	10p12.2	BMI1; COMMD3-BMI1	BMI1 polycomb ring finger oncogene; COMMD3-BMI1 readthrough; NULL	
TC11000477.hg.1	2.26	1.7 × 10^−8^	11q12.1	CNTF; ZFP91; ZFP91-CNTF	ciliary neurotrophic factor; ZFP91 zinc finger protein; ZFP91-CNTF readthrough (NMD candidate); zinc finger protein 91 homolog (mouse); ZFP91-CNTF readthrough (non-protein coding); NULL	
TC11000673.hg.1	1.58	6.5 × 10^−^^13^	11q13.2	RBM14; RBM4; RBM14-RBM4; LOC101059993	RNA binding motif protein 14; RNA binding motif protein 4; RBM14-RBM4 readthrough; uncharacterized LOC101059993; NULL	
TC11002132.hg.1	1.72	7.6 × 10^−8^	11q14.1	NDUFC2-KCTD14; NDUFC2; KCTD14	NDUFC2-KCTD14 readthrough; NADH dehydrogenase (ubiquinone) 1, subcomplex unknown, 2, 14.5kDa; potassium channel tetramerisation domain containing 14; NULL	
TC12001797.hg.1	3.66	1.9 × 10^−12^	12q21.33	POC1B; POC1B-GALNT4; GALNT4	POC1 centriolar protein homolog B (Chlamydomonas); POC1B-GALNT4 readthrough; UDP-N-acetyl-alpha-D-galactosamine:polypeptide N-acetylgalactosaminyltransferase 4 (GalNAc-T4)	
TC13001721.hg.1	1.7	8.3 × 10^−9^	13q33.1	ERCC5; BIVM-ERCC5	excision repair cross-complementing rodent repair deficiency, complementation group 5; BIVM-ERCC5 readthrough; NULL	
TC14001267.hg.1	2.85	5.9 × 10^−10^	14q24.2	SYNJ2BP-COX16; COX16; SYNJ2BP	SYNJ2BP-COX16 readthrough; COX16 cytochrome c oxidase assembly homolog (S. cerevisiae); synaptojanin 2 binding protein	
TC17000082.hg.1	1.83	3 × 10^−11^	17p13.1	RNASEK; C17orf49; RNASEK-C17orf49	ribonuclease, RNase K; chromosome 17 open reading frame 49; RNASEK-C17orf49 readthrough	
TC17002881.hg.1	1.74	1 × 10^−10^	17q21.33	NME2; NME1-NME2	NME/NM23 nucleoside diphosphate kinase 2; NME1-NME2 readthrough; NULL	
TC18001003.hg.1	9.48	3 × 10^−^^10^	18q21.1	SNORD58B; RPL17; RPL17-C18orf32	small nucleolar RNA, C/D box 58B; ribosomal protein L17; RPL17-C18orf32 readthrough	
TC20001752.hg.1	1.72	4.3 × 10^−^^9^	20q13.13	TMEM189; TMEM189-UBE2V1; UBE2V1	transmembrane protein 189; TMEM189-UBE2V1 readthrough; ubiquitin-conjugating enzyme E2 variant 1; NULL	[17]
TC6_apd_hap1000079.hg.1	4.49	1.8 × 10^−^^13^	6p21.33	DDX39B; ATP6V1G2-DDX39B; OTTHUMG00000148789; BAT1	DEAD (Asp-Glu-Ala-Asp) box polypeptide 39B; ATP6V1G2-DDX39B readthrough (NMD candidate); NULL	

## Supplementary Materials

Supplementary materials can be found at https://www.mdpi.com/1422-0067/20/21/5252/s1.

## Abbreviations

Akt	Akt (Protein Kinase B)
AML	Acute Myeloid Leukemia
cis-SAGe	cis-Splicing between Adjacent Genes
Co-TIFE	Co-Transcription-Induced First Exon
CTCF	CCCTC-Binding Factor
DOG	Downstream of Gene Containing Transcript
EST	Expressed Sequence Tag
FuTAG	Fusion Transcript of Adjacent Gene
HGF	Hepatocyte Growth Factor
IP3	Inositol 1,4,5-Trisphosphate
ISP	Intergenic Splicing Pattern
NGS	Next Generation Sequencing
NSCLC	Non-Small Cell Lung Cancer
PAP smear	Papanicolaou Test is a Method of Cervical Screening
SHS	Short Homology Region
ss	Splicing Site
TCGA	The Cancer Genome Atlas
TIC	Transcription-Induced Chimera
TIGF	Transcription Induced Gene Fusion
TNF	Tumor Necrosis Factor
Trans-FT	Trans-Tusion Transcript
