# Supplementary material for: Fusion Transcripts of Adjacent Genes: New Insights into the World of Human Complex Transcripts in Cancer
Source: Int J Mol Sci. 2019 Oct 23;20(21):5252. doi: 10.3390/ijms20215252 (PMC6862657; doi:10.3390/ijms20215252)
Supplement: Supplementary file 1 [file ijms-20-05252-s001.zip › Table S1 oct 13 19.pdf]

**FuTAGs reported in ChiTaRS 3.1 DB (n=25)**	**ConjoinG DB (n=800)**	**Notes**
PPAN-P2RY11	Yes	NM_001040664; NM_001198690
JMJD7-PLA2G4B	No	NM_001198588; NM_005090
NME1-NME2	Yes	NM_001018136; NR_037149
ANKHD1-EIF4EBP3	Yes	NM_020690
PRR5 ARHGAP8	Yes	NM_181334
MEF2B-BORCS8	No	NM_005919
PALM2-AKAP2	Yes	NM_007203; NM_147150
MAGEA10-MAGEA5	Yes	NM_001204811
MROH7-TTC4	Yes	NR_037639; NR_037640; NR_037641
PTGES3L-AARSD1	No	NM_001136042; NM_025267
C8orf44-SGK3	Yes	NM_001204173
FPGT-TNNI3K	Yes	NM_001112808; NM_001199327
ZFP91-CNTF	Yes	NR_024091
STON1-GTF2A1L	Yes	NM_001198593; NM_001198594; NM_172311
SCARNA16-SEC14L1	No	not available on NCBI
SNHG4-MATR3	No	not available on NCBI
KLRC4-KLRK1	Yes	NM_007360
SNHG3-RCC1	No	not available on NCBI
TMEM189-UBE2V1	Yes	NM_199203
PGBD3-ERCC6	No	not available on NCBI
DNAJC25-GNG10	Yes	NM_004125
TRIM6-TRIM34	Yes	NM_001003819
INS-IGF2	Yes	NM_ 001042376; NR_003512
TNFSF12-TNFSF13	Yes	NM_172089
ZNF559-ZNF177	Yes	NM_001172650; NM_001202425

Table S1
